# Major depression and glucidic metabolism: a prospective assessment of response to treatment with antidepressant drugs

**DOI:** 10.1192/j.eurpsy.2026.10593

**Published:** 2026-07-31

**Authors:** R. Pecoraro, R. Ceres, M. De Stefano, C. La Femina, M. Di Mauro, G. Cascino

**Affiliations:** Università degli Studi di Salerno, Salerno, Italy

## Abstract

**Introduction:**

Major depression is one of the leading causes of disability worldwide and is closely associated with physical comorbidities such as cardiometabolic diseases. Comorbidity with type 2 diabetes mellitus (T2DM) is particularly significant. Analysis of the literature shows that adult patients with depression have a 37-60% higher risk of developing T2DM than patients without depressive disorder and that depression in people with T2DM is associated with a more chronic course and a higher frequency of relapses. Insulin resistance (IR), the pathophysiological mechanism underlying T2DM, has been found to be associated with the severity of depressive symptoms in individuals with major depressive episodes. To date, no study has investigated whether the presence of IR/T2DM is associated with a poorer response to psychopharmacological treatment.

**Objectives:**

The aim of the study, therefore, was to identify the impact of IR/DM2 on the response to drug therapy for major depression.

**Methods:**

Patients diagnosed with current major depressive episode not undergoing drug treatment with antidepressants were recruited, and the presence of IR/DM2 was assessed based on HOMA index values. The prospective study lasted 6 months, and all patients recruited in the cross-sectional study were offered SSRI antidepressant therapy. The change in depressive symptoms was assessed after 4, 8, 12, and 18 weeks of drug therapy using the Montgomery Asberg Depression Rating Scale (MADRS). In order to assess the remission rate (MADRS score < 10), Cox regression was used, including IR, gender, age, and severity of depressive symptoms as covariates.

**Results:**

Fifty patients diagnosed with current major depressive episode were recruited, 28 of whom had IR/DM2**.** The groups did not differ significantly in terms of age, gender, and severity of depressive symptoms. The median remission time was 12 weeks for the control group and 24 weeks for the IR/DM2 group. The results suggest that the presence of IR/DM2 is associated with a longer remission time in response to first-line drug treatment (p = 0.003).

**Conclusions:**

In accordance with the initial hypothesis, it emerged that the presence of dysmetabolic conditions, represented by IR/DM2, negatively influences the response to antidepressant drug treatment. These results could support the existence of an immuno-metabolic subtype of major depression, related to the presence of IR/DM2.

**Disclosure of Interest:**

None Declared

